# Association between serum uric acid, hyperuricemia and periodontitis: a cross-sectional study using NHANES data

**DOI:** 10.1186/s12903-023-03320-4

**Published:** 2023-08-30

**Authors:** Jing Xu, Yifan Jia, Zhi Mao, Xiaoxi Wei, Tianyuan Qiu, Min Hu

**Affiliations:** Hospital of Stomatology, Jilin University, Changchun, Jilin Province 130000 China

**Keywords:** Oral health, Periodontal disease, Periodontal health, Hyperuricemia, Uric acid

## Abstract

**Objectives:**

Diabetes and other metabolic diseases have been linked to the development of periodontitis, but little research has been done to determine whether serum uric acid (SUA) levels and hyperuricemia play a role. This study aimed to investigate the relationship between SUA, hyperuricemia, and periodontitis.

**Methods:**

Using data from the National Health and Nutrition Examination Survey (NHANES) 2011–2014, we created a nationally representative data set. We used multivariable logistic regression models to assess the relationship between SUA, hyperuricemia, and periodontitis and presented odds ratios (OR) in women and men, respectively.

**Results:**

In women, adjusted multivariable regression models showed that SUA (4.1–4.3mg/dl) was associated with higher odds of periodontitis (OR = 1.43; 95% confidence interval (CI):1.0 ~ 2.03, *p* = 0.047) with SUA (≤ 3.3mg/dl) as reference. The risk of periodontitis tended to increase slightly but insignificantly with increasing SUA levels, and the adverse effects occurred only when SUA increased to a certain level, and then reached a plateau. In men, the adjusted OR values for SUA (4.9–5.2mg/dl), SUA (5.3–5.5mg/dl), SUA (5.9–6.2mg/dl), and SUA (6.3–6.5mg/dl) were 0.66 (95% CI: 0.45 ~ 0.96, *p* = 0.029), 0.58 (95% CI: 0.40 ~ 0.85, *p* = 0.006), 0.67(95% CI: 0.47 ~ 0.97, *p* = 0.035), and 0.67 (95% CI: 0.45 ~ 0.99, *p* = 0.043), respectively, with SUA (≤ 4.3mg/dl) as reference. The elevated SUA levels are protective against periodontitis, but there is a range within which the risk of periodontitis decreases, followed by a non-significant tendency to increase.

**Conclusions:**

The levels of SUA that are linked to the risk of periodontitis. Future prospective longitudinal studies and strategies are required to further confirm whether controlled SUA treatment is an effective adjunct to systematic periodontal therapy and whether SUA can be used as a diagnostic biomarker to assess the risk or progression of periodontitis.

**Supplementary Information:**

The online version contains supplementary material available at 10.1186/s12903-023-03320-4.

## Introduction

Serum uric acid (SUA) is a purine nucleotide metabolite derived from exogenous dietary and endogenous nucleic acids. Normal SUA levels are 1.5–6 mg/dl in women and 2.5–7 mg/dl in men. Although certain ranges of SUA are considered a beneficial antioxidant [1], excessive SUA, or hyperuricemia, is an independent risk factor for many diseases (metabolic syndrome, diabetes, hypertension, cardiovascular events, kidney disease, etc.) [2]. Currently, hyperuricemia is the second most common metabolic disease in the world, affecting 21.7% of the male population and 14.4% of the female population [3].

Periodontitis is a common microbially-induced chronic inflammatory disease characterized by irreparable loss of alveolar bone and periodontal ligament, which can lead to tooth loss. The disease is also influenced by various factors such as diabetes, osteoporosis, inflammatory bowel disease, obesity, and smoking [4]. In 2010, approximately 10% of people worldwide had severe periodontitis, making it the sixth most common health condition [5]. Between 2009 and 2012, 46% of American adults had periodontitis, with 8.9% having severe periodontitis [6]. Furthermore, periodontitis extends beyond the oral cavity and acts as an influential and correlated factor with systemic diseases such as diabetes, Alzheimer's disease, chronic obstructive pulmonary disease, and cardiovascular disease [7, 8].

The direct and indirect relationship between hyperuricemia and periodontitis is receiving more attention [1]. Sato et al. showed that elevated SUA may be a cause of alveolar bone destruction in obesity-related periodontitis. Periodontitis induction caused more severe bone destruction in hyperuricemia mice than in normouricemia mice, and the worsened bone destruction was completely abrogated by allopurinol, a xanthine oxidase inhibitor [9]. Some researchers speculated that hyperuricemia and periodontitis may influence and even promote each other based on periodontal pathogens and immune-metabolic mechanisms (impaired immune response, oxidative stress, pathological bone remodeling and ecological dysregulation, etc.) [1, 3, 10, 11]. Soluble SUA may induce a sterile immunoinflammatory response via cross-inflammatory signaling pathways, resulting in more severe destruction of periodontal supporting tissues [12]. In contrast, other researchers have suggested that hyperuricemia and high levels of SUA may have a beneficial effect on periodontitis [13].

In addition to understanding the main causes, pathogenesis and treatment of periodontitis, reducing the associated risk factors is equally important for the successful prevention and treatment of periodontitis. The relationship between SUA, hyperuricemia, and periodontitis is unclear, and only a few experimental research and epidemiological studies investigated it. Given the high prevalence of hyperuricemia and periodontitis, SUA may influence the global burden of periodontal disease directly or indirectly, and clarification of the relationship is needed. We conducted a large-scale clinical cross-sectional study using the National Health and Nutrition Examination Survey (NHANES) database to investigate the association between SUA, hyperuricemia, and periodontitis.

## Methods

### Data

**Fig. 1:**
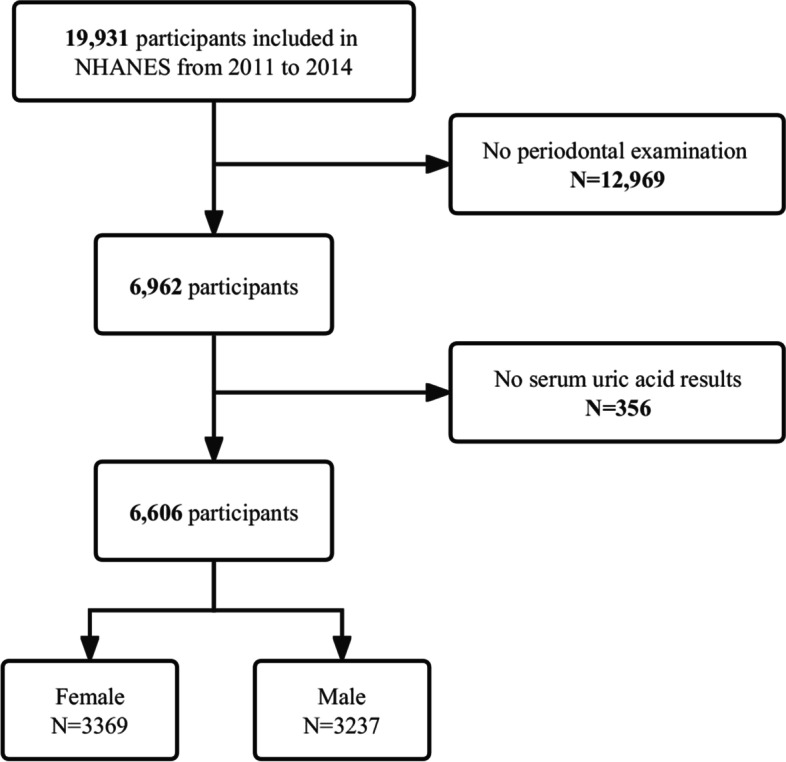
Study flow diagram

This cross-sectional study used NHANES data from 2011 to 2014, performed by the Centers for Disease Control and Prevention. The NHANES gathers demographic and detailed health information through home visits, screening, and laboratory testing by a mobile examination center (MEC). The NHANES was authorized by the National Center for Health Statistics (NCHS) Ethics Review Committee, and all participants completed written informed consent forms before participation. The secondary analysis did not require additional Institutional Review Board approval. In total, 19,931 participants completed the interview (Fig. 1). A total of 6,962 adults aged 30–80 with periodontal exams constituted the study sample and we further excluded those who had not yet completed blood laboratory tests for SUA (*n* = 356). Ultimately, this cross-sectional study included 6,606 participants in the analysis. For all analyses, the percentages of missing values of covariates were lower than 8%. We used multiple imputations, based on 5 replications and a chained equation approach method in the R MI procedure, to maximize statistical power and minimize bias that might occur to account for missing data.

### Study variables

#### Primary outcome: periodontitis

To determine periodontal status, licensed calibrated dental examiners performed a full-mouth periodontal examination. The full-mouth periodontal examination includes an evaluation of gingival recession and pocket depth measures in a MEC. Periodontal exams were performed following the NHANES examination protocol by trained dentists [14]. Clinical attachment loss (AL) and probing depth (PD) from four inter-proximal sites per tooth (mesio-buccal, disto-buccal, mesio-lingual, disto-lingual) on all teeth except third molars were used to define periodontitis [15]. For mild periodontitis, the following definition was proposed: ≥ 2 interproximal sites with AL ≥ 3 mm and ≥ 2 interproximal sites with PD ≥ 4 mm (not on the same tooth), or one site with PD ≥ 5 mm. For moderate periodontitis, the following definition was proposed: ≥ 2 interproximal sites with AL ≥ 4 mm (not on the same tooth), or ≥ 2 interproximal sites with PD ≥ 5 mm (not on the same tooth). For severe periodontitis, the following definition was proposed: ≥ 2 interproximal sites with AL ≥ 6 mm (not on the same tooth) and ≥ 1 interproximal site with PD ≥ 5 mm. Periodontitis status was classified into "yes" (mild, moderate, and severe) and "no".

#### Primary exposure: SUA and hyperuricemia

Serum specimens are processed, stored, and shipped to the Collaborative Laboratory Services for analysis. Vials are stored under appropriate frozen (–30°C) conditions until they are shipped to National Center for Environment Health for testing. The DxC800 uses a timed endpoint method to measure the concentration of SUA in serum, plasma or urine. SUA is oxidized by uricase to produce allantoin and hydrogen peroxide. The hydrogen peroxide reacts with 4-amino antipyrine (4-AAP) and 3, 5-dichloro-2-hydroxybenzene sulfonate (DCHBS) in a reaction catalyzed by peroxidase to produce a colored product. The system monitors the change in absorbance at 520 nm at a fixed time interval. The change in absorbance is directly proportional to the concentration of SUA in the sample. SUA data analysis with continuous variables and deciles. This study defined hyperuricemia as being above 7.0 mg/dL in the male population and above 6.0 mg/dL in the female population [16].

#### Other covariates

A variety of potential covariates were assessed according to the literature [13, 17], including age; race/ethnicity; education; marital status; income-poverty ratio (PIR); alcohol status; smoking status; dietary fiber consumption; total fat consumption; body mass index (BMI); gout; congestive heart failure; coronary heart disease; angina; stroke; weak/failing kidneys; diabetes; hypertension and dental visit. Race/ethnicity was categorized as Mexican American, other Hispanic, non-Hispanic white, non-Hispanic black, non-Hispanic Asian, or other race. The educational level was classified as below high school, high school, or above. Marital status was classified as married, living with a partner, or living alone. According to a US government population report, family income was categorized into three groups by the PIR: low (≤ 1), medium (1–3), and high (> 3). Alcohol status was classified as "yes" or "no" based on whether or not the individual consumed more than 12 alcoholic drinks per year. Smoking status was classified as "yes" or "no" depending on whether more than 100 cigarettes had been smoked. A dietary recall interview preceded an interview at MEC to obtain participants’ 24-h nutritional information, including dietary fiber and total fat. BMI was classified as underweight or normal weight, overweight, or obese based on BMI < 25, 25–30, or > 30, respectively. The determination of previous disease (gout, congestive heart failure, coronary heart disease, angina, stroke, weak/failing kidneys, diabetes, and hypertension) was based on the inquiry in the questionnaire of whether the doctor had been informed of the condition in the past. The dental visit was based on whether had a professional oral exam at the dental office within one year. If a given covariate resulted in a change in effect estimate of more than 10% or was significantly associated with periodontitis, the variable was chosen as a confounder [18].

### Analysis

Categorical variables were represented by proportions (%) while continuous variables were described by the mean (standard deviation, SD) or median (interquartile range, IQR), as appropriate. Due to different SUA metabolisms in different sexes, analyses were performed separately by sex. To compare the differences across groups, t-test (normal distribution), Wilcoxon rank-sum test (skewed distribution), and chi-square tests (categorical variables) were undertaken. Logistic regression models were used to determine the odds ratios (OR) and 95 percent confidence intervals (95% CIs) for the association between SUA, hyperuricemia and periodontitis. Model I was adjusted for socio-demographic characteristics, including age, race/ethnicity, PIR, education and marital status. Model II was adjusted for sociodemographic characteristics, smoking status, alcohol status, BMI, dietary fiber, and total fat. Model III was adjusted for sociodemographic characteristics, smoking status, alcohol status, BMI, dietary fiber, total fat, comorbidities (gout, congestive heart failure, coronary heart disease, angina, stroke, weak/failing kidneys, diabetes, hypertension) and dental visit.

Restricted cubic spline (RCS) regression was performed with 4 knots at the 5th, 35th, 65th, and 95th percentiles of SUA to assess linearity and examine the dose–response curve between SUA and periodontitis after adjusting for the variables in Model III in the male and female populations, respectively. Furthermore, subgroup analyses were also conducted, including the following stratifications: age, marital status, PIR and BMI. Heterogeneity between subgroups was assessed by multivariate logistic regression, and interactions between subgroups and SUA were examined by likelihood ratio testing. All analyses were performed with the statistical software package R 3.3.2 (http://www.R-project.org, The R Foundation, accessed 24 January 2023) and Free Statistics software version 1.7. A *p*-value of < 0.05 was considered significant using a two-tailed test. This study followed the Strengthening the Reporting of Observational Studies in Epidemiology (STROBE) guidelines for cross-sectional studies.

## Results

### Baseline characteristics of those with and without periodontitis by sex

The baseline characteristics of females are shown in Table 1. The average age of the female population was 51.8 (14.1) years. Compared with females without periodontitis, those with periodontitis had higher SUA levels and a higher prevalence of hyperuricemia. Table 2 shows the baseline characteristics of the male population. Males were aged 52.0 (14.4) on average. There was no significant difference in the prevalence of hyperuricemia or SUA levels between those with and without periodontitis.

**Table 1:** Characteristics of the 3,369 participants with and without periodontitis in the female population

Variables	Total (*n* = 3369)	No periodontitis (*n* = 1876)	Periodontitis (*n* = 1493)	*P*-value
SUA (mg/dl)	4.9 ± 1.3	4.8 ± 1.2	5.0 ± 1.4	< 0.001^**^
Hyperuricemia, n (%)	539 (16.0)	258 (13.8)	281 (18.8)	< 0.001^**^
Age (years)	51.8 ± 14.1	48.8 ± 13.6	55.5 ± 13.8	< 0.001^**^
Race/ethnicity, n (%)				< 0.001^**^
Mexican American	402 (11.9)	183 (9.8)	219 (14.7)	
Other Hispanic	343 (10.2)	190 (10.1)	153 (10.2)	
Non-Hispanic White	1376 (40.8)	873 (46.5)	503 (33.7)	
Non-Hispanic Black	724 (21.5)	337 (18)	387 (25.9)	
Non-Hispanic Asian	440 (13.1)	241 (12.8)	199 (13.3)	
Other Race	84 (2.5)	52 (2.8)	32 (2.1)	
Education, n (%)				< 0.001^**^
< High school	653 (19.4)	252 (13.4)	401 (26.9)	
High school	690 (20.5)	319 (17)	371 (24.8)	
> High school	2026 (60.1)	1305 (69.6)	721 (48.3)	
Marital status, n (%)				< 0.001^**^
Married or living with partner	1387 (41.2)	665 (35.4)	722 (48.4)	
Living alone	1982 (58.8)	1211 (64.6)	771 (51.6)	
PIR, n (%)				< 0.001^**^
≤ 1	724 (21.5)	305 (16.3)	419 (28.1)	
1–3	1298 (38.5)	637 (34)	661 (44.3)	
> 3	1347 (40.0)	934 (49.8)	413 (27.7)	
Alcohol status, n (%)	2049 (60.8)	1232 (65.7)	817 (54.7)	< 0.001^**^
Smoking status, n (%)	1143 (33.9)	566 (30.2)	577 (38.6)	< 0.001^**^
Dietary fiber (gm)	14.2 (9.4, 20.6)	14.7 (9.9, 21.1)	13.4 (9.0, 19.9)	< 0.001^**^
Total fat (gm)	64.1 (43.4, 89.8)	66.5 (46.0, 91.3)	60.4 (40.4, 88.0)	< 0.001^**^
BMI (kg/m^2^), n (%)				< 0.001^**^
< 25	984 (29.2)	605 (32.2)	379 (25.4)	
25–30	971 (28.8)	544 (29)	427 (28.6)	
> 30	1414 (42.0)	727 (38.8)	687 (46)	
Gout, n (%)	75 (2.2)	27 (1.4)	48 (3.2)	< 0.001^**^
Congestive heart failure, n (%)	86 (2.6)	27 (1.4)	59 (4)	< 0.001^**^
Coronary heart disease, n (%)	65 (1.9)	21 (1.1)	44 (2.9)	< 0.001^**^
Angina, n (%)	62 (1.8)	22 (1.2)	40 (2.7)	0.001^**^
Stroke, n (%)	98 (2.9)	40 (2.1)	58 (3.9)	0.003^**^
Weak/failing kidneys, n (%)	109 (3.2)	38 (2)	71 (4.8)	< 0.001^**^
Diabetes, n (%)	413 (12.3)	160 (8.5)	253 (16.9)	< 0.001^**^
Hypertension, n (%)	1300 (38.6)	612 (32.6)	688 (46.1)	< 0.001^**^
Dental visit, n (%)	2100 (62.3)	1313 (70)	787 (52.7)	< 0.001^**^

*Abbreviations*: *SUA* serum uric acid, *PIR* income-poverty ratio, *BMI* body mass index

^*^p < 0.05; **p < 0.01

**Table 2:** Characteristics of the 3,237 participants with and without periodontitis in the male population

Variables	Total (*n* = 3237)	No periodontitis (*n* = 1246)	Periodontitis (*n* = 1991)	*P*-value
SUA (mg/dl)	6.0 ± 1.3	6.1 ± 1.3	6.0 ± 1.3	0.051
Hyperuricemia, n (%)	635 (19.6)	248 (19.9)	387 (19.4)	0.745
Age (years)	52.0 ± 14.4	47.4 ± 13.6	54.8 ± 14.1	< 0.001^**^
Race/ethnicity, n (%)				< 0.001^**^
Mexican American	414 (12.8)	111 (8.9)	303 (15.2)	
Other Hispanic	287 (8.9)	94 (7.5)	193 (9.7)	
Non-Hispanic White	1335 (41.2)	648 (52)	687 (34.5)	
Non-Hispanic Black	697 (21.5)	186 (14.9)	511 (25.7)	
Non-Hispanic Asian	418 (12.9)	171 (13.7)	247 (12.4)	
Other Race	86 (2.7)	36 (2.9)	50 (2.5)	
Education, n (%)				< 0.001^**^
< High school	719 (22.2)	146 (11.7)	573 (28.8)	
High school	728 (22.5)	204 (16.4)	524 (26.3)	
> High school	1790 (55.3)	896 (71.9)	894 (44.9)	
Marital status, n (%)				0.002^**^
Married or living with partner	930 (28.7)	319 (25.6)	611 (30.7)	
Living alone	2307 (71.3)	927 (74.4)	1380 (69.3)	
PIR, n (%)				< 0.001^**^
≤ 1	597 (18.4)	137 (11)	460 (23.1)	
1–3	1238 (38.2)	386 (31)	852 (42.8)	
> 3	1402 (43.3)	723 (58)	679 (34.1)	
Alcohol status, n (%)	2730 (84.3)	1046 (83.9)	1684 (84.6)	0.63
Smoking status, n (%)	1712 (52.9)	517 (41.5)	1195 (60)	< 0.001^**^
Dietary fiber (gm)	17.4 (11.3, 25.5)	18.2 (12.3, 26.3)	16.7 (10.6, 25.1)	< 0.001^**^
Total fat (gm)	82.2 (55.5, 114.5)	86.6 (61.9, 117.5)	78.7 (51.7, 112.8)	< 0.001^**^
BMI (kg/m^2^), n (%)				0.164
< 25	836 (25.8)	299 (24)	537 (27)	
25–30	1300 (40.2)	516 (41.4)	784 (39.4)	
> 30	1101 (34.0)	431 (34.6)	670 (33.7)	
Gout, n (%)	192 (5.9)	65 (5.2)	127 (6.4)	0.173
Congestive heart failure, n (%)	78 (2.4)	17 (1.4)	61 (3.1)	0.002^**^
Coronary heart disease, n (%)	131 (4.0)	41 (3.3)	90 (4.5)	0.084
Angina, n (%)	76 (2.3)	24 (1.9)	52 (2.6)	0.21
Stroke, n (%)	86 (2.7)	21 (1.7)	65 (3.3)	0.007^**^
Weak/failing kidneys, n (%)	104 (3.2)	29 (2.3)	75 (3.8)	0.024^*^
Diabetes, n (%)	436 (13.5)	105 (8.4)	331 (16.6)	< 0.001^**^
Hypertension, n (%)	1226 (37.9)	398 (31.9)	828 (41.6)	< 0.001^**^
Dental visit, n (%)	1812 (56.0)	853 (68.5)	959 (48.2)	< 0.001^**^

*Abbreviations*: *SUA* serum uric acid, *PIR* income-poverty ratio, *BMI* body mass index

^*^p < 0.05; **p < 0.01

### Relationship between SUA, hyperuricemia and periodontitis by sex

**Fig. 2:**
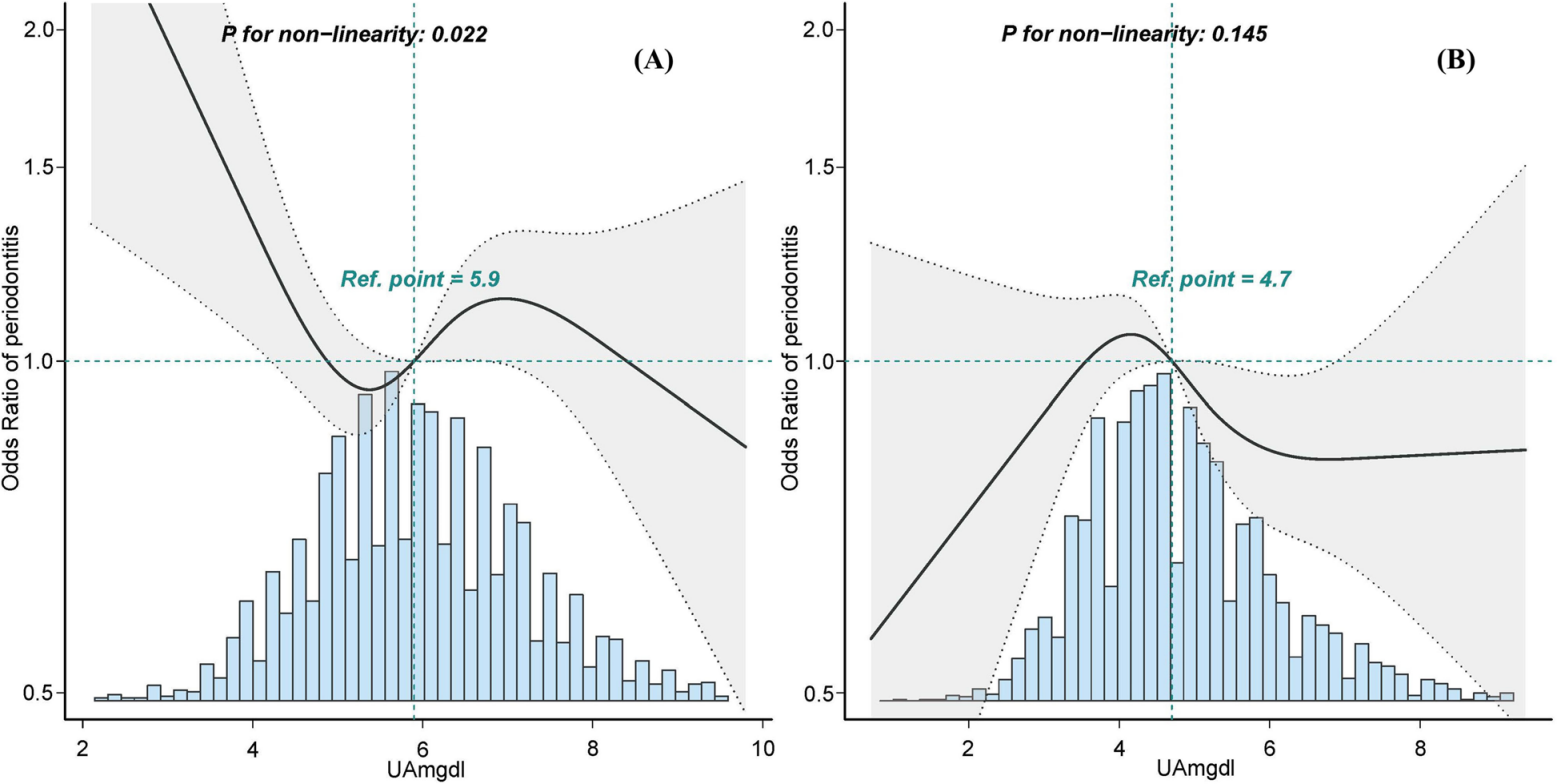
Dose–response relationship between the SUA and periodontitis in the female population (**A**) and the male population (**B**) adjusted for model III. Solid and dashed lines represent the predicted value and 95%confidence intervals

After adjustment for potential confounders, neither hyperuricemia nor continuous SUA was associated with periodontitis (Tables 3 and 4). In women, the fully adjusted OR for SUA and periodontitis in D4 (4.1–4.3mg/dl) was 1.43 (95% CI: 1.0 ~ 2.03, *p* = 0.047) with D1 (≤ 3.3mg/dl) as reference (Table 3). Combining the dose–response relationship between SUA and periodontitis in women (Fig. 2A), we found that the risk of periodontitis tended to increase slightly but insignificantly with increasing SUA levels, and the adverse effects occurred only when SUA increased to a certain level, and then reached a plateau. Table 4 shows the relationship between SUA and periodontitis in men. When SUA was analyzed using deciles, the fully adjusted OR values in D3 (4.9–5.2mg/dl), D4 (5.3–5.5mg/dl), D6 (5.9–6.2mg/dl), and D7 (6.3–6.5mg/dl) were 0.66 (95% CI: 0.45 ~ 0.96, *p* = 0.029), 0.58 (95% CI: 0.40 ~ 0.85, *p* = 0.006), 0.67(95% CI: 0.47 ~ 0.97, *p* = 0.035), and 0.67 (95% CI: 0.45 ~ 0.99,* p* = 0.043), respectively, with D1 (≤ 4.3mg/dl) as reference. The association between SUA and periodontitis in men was not linear, but rather a curve (non-linear, *p* = 0.022) in RCS (Fig. 2B). The results suggest that elevated SUA levels are protective against periodontitis, but there is a range within which the risk of periodontitis decreases, followed by a slight and non-significant tendency to increase.

**Table 3:** Logistic regression to determine the odds of periodontitis presence by hyperuricemia or SUA in the female population

Variable	Non-adjusted Model		Model I		Model II		Model III	
	**OR (95%CI)**	***P*-value**	**OR (95%CI)**	***P*-value**	**OR (95%CI)**	***P*-value**	**OR (95%CI)**	***P*-value**
Hyperuricemia	1.45 (1.21 ~ 1.75)	< 0.001^**^	1.03 (0.84 ~ 1.26)	0.794	0.9 (0.73 ~ 1.11)	0.332	0.86 (0.69 ~ 1.07)	0.169
SUA, mg/dl	1.14 (1.08 ~ 1.21)	< 0.001^**^	1.02 (0.96 ~ 1.08)	0.524	0.97 (0.91 ~ 1.03)	0.348	0.96 (0.9 ~ 1.03)	0.226
SUA, deciles								
D1(≤ 3.3mg/dl)	1(Ref)		1(Ref)		1(Ref)		1(Ref)	
D2(3.4–3.7mg/dl)	1.25 (0.9 ~ 1.73)	0.186	1.35 (0.94 ~ 1.92)	0.101	1.31 (0.92 ~ 1.87)	0.139	1.34 (0.94 ~ 1.93)	0.108
D3(3.8–4.0mg/dl)	1.38 (0.99 ~ 1.91)	0.055	1.47 (1.03 ~ 2.09)	0.033^*^	1.38 (0.97 ~ 1.97)	0.077	1.4 (0.98 ~ 2)	0.068
D4(4.1–4.3mg/dl)	1.41 (1.03 ~ 1.94)	0.033^*^	1.44 (1.02 ~ 2.03)	0.039^*^	1.37 (0.97 ~ 1.94)	0.077	1.43 (1 ~ 2.03)	0.047^*^
D5(4.4–4.6mg/dl)	1.27 (0.93 ~ 1.74)	0.133	1.25 (0.89 ~ 1.75)	0.204	1.15 (0.82 ~ 1.63)	0.414	1.17 (0.83 ~ 1.66)	0.373
D6(4.7–4.9mg/dl)	1.53 (1.11 ~ 2.1)	0.009^**^	1.46 (1.03 ~ 2.07)	0.032^*^	1.31 (0.92 ~ 1.87)	0.129	1.35 (0.95 ~ 1.92)	0.099
D7(5.0–5.3mg/dl)	1.42 (1.04 ~ 1.93)	0.027^*^	1.25 (0.9 ~ 1.75)	0.189	1.13 (0.8 ~ 1.59)	0.48	1.16 (0.82 ~ 1.64)	0.386
D8(5.4–5.7mg/dl)	1.38 (0.99 ~ 1.91)	0.056	1.09 (0.76 ~ 1.55)	0.647	0.95 (0.66 ~ 1.37)	0.775	0.97 (0.67 ~ 1.41)	0.891
D9(5.8–6.5mg/dl)	1.48 (1.08 ~ 2.02)	0.014^*^	1.15 (0.82 ~ 1.62)	0.411	0.96 (0.68 ~ 1.37)	0.834	1.01 (0.71 ~ 1.44)	0.969
D10(≥ 6.6mg/dl)	2.28 (1.66 ~ 3.13)	< 0.001^**^	1.5 (1.06 ~ 2.13)	0.021^*^	1.19 (0.82 ~ 1.71)	0.355	1.14 (0.78 ~ 1.65)	0.496

1 (Ref) = reference; omitted for collinearity

*Abbreviations*: *D* deciles, *OR* odds ratio, *CI* confidence interval, *SUA* serum uric acid

Model I adjusts for age, race/ethnicity, income-poverty ratio (PIR), education and marital status

Model II adjusts for Model I + smoking status, alcohol status, BMI, dietary fiber and total fat

Model III adjusts for Model I + Model II + gout, congestive heart failure, coronary heart disease, angina, stroke, weak/failing kidneys, diabetes, hypertension and dental visit

^*^p < 0.05; **p < 0.01

**Table 4:** Logistic regression to determine the odds of periodontitis presence by hyperuricemia or SUA in the male population

Variable	Non-adjusted Model		Model I		Model II		Model III	
	**OR (95%CI)**	***P*-value**	**OR (95%CI)**	***P*-value**	**OR (95%CI)**	***P*-value**	**OR (95%CI)**	***P*-value**
Hyperuricemia	0.97 (0.81 ~ 1.16)	0.745	1.05 (0.86 ~ 1.29)	0.606	1.05 (0.85 ~ 1.29)	0.673	1.06 (0.85 ~ 1.31)	0.608
SUA, mg/dl	0.95 (0.9 ~ 1)	0.051	0.98 (0.92 ~ 1.04)	0.447	0.97 (0.91 ~ 1.04)	0.376	0.98 (0.91 ~ 1.04)	0.467
SUA, deciles								
D1(≤ 4.3mg/dl)	1(Ref)		1(Ref)		1(Ref)		1(Ref)	
D2(4.4–4.8mg/dl)	0.81 (0.57 ~ 1.14)	0.225	0.82 (0.56 ~ 1.21)	0.32	0.82 (0.56 ~ 1.2)	0.305	0.83 (0.56 ~ 1.22)	0.349
D3(4.9–5.2mg/dl)	0.58 (0.41 ~ 0.8)	0.001^**^	0.66 (0.46 ~ 0.95)	0.025^*^	0.67 (0.46 ~ 0.97)	0.034^*^	0.66 (0.45 ~ 0.96)	0.029^*^
D4(5.3–5.5mg/dl)	0.46 (0.33 ~ 0.65)	< 0.001^**^	0.57 (0.39 ~ 0.82)	0.003^**^	0.56 (0.39 ~ 0.82)	0.003^**^	0.58 (0.4 ~ 0.85)	0.006^**^
D5(5.6–5.8mg/dl)	0.6 (0.43 ~ 0.84)	0.003^**^	0.7 (0.49 ~ 1.02)	0.062	0.72 (0.5 ~ 1.05)	0.09	0.75 (0.51 ~ 1.09)	0.129
D6(5.9–6.2mg/dl)	0.54 (0.39 ~ 0.75)	< 0.001^**^	0.64 (0.45 ~ 0.91)	0.012^*^	0.66 (0.46 ~ 0.94)	0.022^*^	0.67 (0.47 ~ 0.97)	0.035^*^
D7(6.3–6.5mg/dl)	0.62 (0.44 ~ 0.87)	0.007^**^	0.64 (0.44 ~ 0.94)	0.024^*^	0.64 (0.43 ~ 0.94)	0.023^*^	0.67 (0.45 ~ 0.99)	0.043^*^
D8(6.6–6.9mg/dl)	0.69 (0.49 ~ 0.96)	0.03^*^	0.81 (0.56 ~ 1.17)	0.266	0.79 (0.54 ~ 1.15)	0.218	0.8 (0.54 ~ 1.17)	0.252
D9(7.0–7.7mg/dl)	0.62 (0.45 ~ 0.85)	0.004^**^	0.8 (0.56 ~ 1.14)	0.219	0.8 (0.56 ~ 1.15)	0.23	0.83 (0.57 ~ 1.19)	0.31
D10(≥ 7.8mg/dl)	0.66 (0.46 ~ 0.93)	0.017^*^	0.74 (0.51 ~ 1.09)	0.128	0.74 (0.5 ~ 1.09)	0.127	0.75 (0.5 ~ 1.13)	0.169

(Ref) = reference; omitted for collinearity

*Abbreviations*: *D* deciles, *OR* odds ratio, *CI* confidence interval, *SUA* serum uric acid

Model I adjusts for age, race/ethnicity, income-poverty ratio (PIR), education and marital status

Model II adjusts for Model I + smoking status, alcohol status, BMI, dietary fiber and total fat

Model III adjusts for Model I + Model II + gout, congestive heart failure, coronary heart disease, angina, stroke, weak/failing kidneys, diabetes, hypertension and dental visit

^*^p < 0.05; **p < 0.01

### Subgroup and sensitivity analyses

Stratified analyses were performed in several subgroups to assess potential effect modifications on the association between SUA and periodontitis in females and males, respectively. Apart from interaction with age in the male population, no significant interactions were found in any of the subgroups after stratification by age, marital status, PIR and BMI (Supplementary Figure S1, Supplementary Figure S2). Overall, the trends and core results were consistent across subgroups, although some were not statistically significant, possibly due to the small sample size after splitting. After excluding missing covariates, the association between SUA, hyperuricemia and periodontitis presented similar trends, although the degree of association became slightly weaker.

## Discussion

Compared with previous studies, this study divided the total population into two groups: men and women, due to gender differences in SUA levels and conditions for determining hyperuricemia. Changes in SUA levels in patients with periodontitis or after periodontal treatment have shown that elevated SUA levels may be positively correlated with the severity of periodontitis [19, 20]. Women with periodontitis had higher SUA levels and a higher prevalence of hyperuricemia in our population, which is consistent with previous observational and interventional studies [12, 19–21]. This may be due to the higher prevalence of kidney disease and diabetes in women with periodontitis. Chronic kidney disease and diabetes can manifest as decreased glomerular filtration rate, resulting in decreased SUA excretion and higher SUA levels or hyperuricemia [3, 22]. In the female population, the univariate logistic regression results of this study indicated that the risk of periodontitis increased with elevated SUA levels and the presence of hyperuricemia. Although the association between hyperuricemia and periodontitis lost statistical significance after adjusting for potential confounders, we found that there was still a trend toward increased risk of periodontitis with elevated SUA levels within a certain range, although the significance disappeared after this.

In men, there was no significant difference in SUA levels and the prevalence of hyperuricemia between those with and without periodontitis. This may be related to the higher prevalence of periodontitis and hyperuricemia in male patients and the presence of more susceptibility factors to elevated SUA levels in men's lifestyles, such as alcohol consumption. Multivariate logistic regression results suggest that elevated SUA levels are protective against periodontitis, but there is a range within which the risk of periodontitis decreases, after which the protection disappears. The source of this difference between men and women is not yet known, but based on previous studies, we can further discuss the phenomenon of association in men. Byun et al. suggested that the adjusted OR of hyperuricemia for periodontitis was 0.89 (95% CI: 0.81 ~ 0.96, *p* = 0.005) and that elevated SUA levels may have a positive effect on periodontitis. However, this study did not indicate the range of SUA levels that are beneficial for periodontal health [13]. Our results appear to complement the previous findings, but we emphasize that SUA levels of 4.9–6.5 mg/dl with ≤ 4.3mg/dl as a reference may be the ideal range to effectively reduce the risk of periodontitis, rather than a hyperuricemia state. In addition to grouping by sex, the difference in results with Byun may be related to the fact that he does not adequately adjust for the metabolic comorbidities of periodontitis and hyperuricemia as confounding factors [13].

Although the underlying mechanism of the association between SUA and periodontitis remains to be investigated, our findings in men are biologically plausible based on the following evidence. Due to the antioxidant properties of SUA, elevated SUA levels within the normal range are considered a protective factor for maintaining bone mineral density and resisting bone resorption [23–25]. As osteoporosis is a risk factor for periodontitis [26, 27], high SUA levels may reduce the risk of developing periodontitis by inhibiting osteoporosis development. Furthermore, we concluded that this protective role was no longer statistically significant when the SUA levels exceeded 6.5 mg/dl or became hyperuricemia. This finding is consistent with the following conclusion: SUA levels above normal are a risk factor for osteoporosis and fractures, as oxidative stress and inflammatory cytokines may increase bone resorption and decrease bone formation [1, 23, 28, 29]. In addition, hyperuricemia is an independent risk factor for many diseases associated with periodontitis (metabolic syndrome, diabetes, hypertension, cardiovascular events, kidney disease, etc.) [11]. The protective effects of elevated SUA at this time are offset by the tendency to increase the risk of periodontitis. Therefore, in men with periodontitis, proper control of SUA is not only beneficial for their general health but also may help to reduce the risk of periodontitis.

The treatment of periodontitis is complex and in addition to regular periodontal maintenance (scaling, scraping), the current treatment philosophy emphasizes self-management through patient education, which mainly includes self-monitoring, lifestyle interventions, early detection and avoidance of risk factors. Establishing a relationship between periodontitis and metabolic disease will facilitate the development of periodontitis treatment and prevention. There are some common risk factors for self-care of SUA levels and periodontal health, and individuals who successfully control their SUA levels may also tend to have better periodontal control. According to our findings, adequate control of SUA levels is beneficial in reducing the risk of periodontitis. As a next step, we recommend that future research should conduct longitudinal studies to confirm whether controlled SUA treatment is an effective adjunct to systematic periodontal therapy and whether SUA can be used as a diagnostic biomarker to assess the risk or progression of periodontitis. This has important implications for the lifestyle, diet and disease prevention of periodontitis patients. In addition, the mechanism underlying the relationship between SUA, hyperuricemia and periodontitis differs between men and women requires further research.

In the present study, we analyzed a representative sample of multiracial populations for better generalizability to the United States population. In addition, participants were evaluated and operated by trained staff, and interviews were conducted following standardized procedures and strict quality control to obtain examination data and laboratory data, which improved the accuracy and validity of the data. Given the gender differences in SUA levels and the determination of hyperuricemia, the total population was divided into two groups of men and women. In the data analysis, we adjusted for a considerable number of potential confounding variables. Such a large sample size enabled us to carry out further subgroup and piecewise model analyses. In addition, the potential association between SUA, hyperuricemia and periodontitis may help researchers to conduct further randomized controlled trials or cohort studies in related aspects.

But some limitations need to be considered. First, this was a cross-sectional design, and the direction of the association between SUA and periodontitis was difficult to explain as causality. Second, although we adjusted for more than a dozen major covariates in the analysis, we may have missed some possible residual confounders that were not considered in the model design or for which there was insufficient information in the database to allow proper adjustment. Third, the data source of the NHANES database is the US population. Therefore, the results should be extrapolated with caution to other populations in different countries.

## Conclusion

The treatment of periodontal disease is based on scaling, smoothing and polishing, the removal of plaque retentive factors and correct hygiene by the patient. Likewise, systemic diseases and habits must be located so that the treatment is completely successful. Combining the global burden of periodontitis with SUA has important clinical and public health implications for dental teams, and the role of SUA in periodontitis should be emphasized. Individuals with periodontitis require special attention to lifestyle (diet, smoking, drinking, exercise, etc.), weight control, treatment and prevention of metabolic and cardiovascular diseases, and the use of medications to control their SUA levels, which may help reduce the risk of periodontitis. From a medical and therapeutic point of view, further studies are needed to understand whether the association between periodontitis and SUA is merely correlative or originates from a causal interaction, thus providing a reference for periodontal and general health.

### Supplementary Information

**Additional file 1: Supplementary Figure 1.** Subgroup analyses of SUA and periodontitis in the female population. Except for the stratification component itself, each stratification factor was adjusted for all other variables (age, race/ethnicity, education, marital status, income-poverty ratio (PIR), alcohol status, smoking status, dietary fiber, total fat, body mass index (BMI), gout, congestive heart failure, coronary heart disease, angina, stroke, weak/failing kidneys, diabetes, hypertension and dental visit).

**Additional file 2: Supplementary Figure 2.** Subgroup analyses of SUA and periodontitis in the male population. Except for the stratification component itself, each stratification factor was adjusted for all other variables (age, race/ethnicity, education, marital status, income-poverty ratio (PIR), alcohol status, smoking status, dietary fiber, total fat, body mass index (BMI), gout, congestive heart failure, coronary heart disease, angina, stroke, weak/failing kidneys, diabetes, hypertension and dental visit).

**Additional file 3: Supplementary Table 1.** Baseline characteristics of the study population by sex. 

**Additional file 4: Supplementary Table 2.** Results of univariate analysis of periodontitis in women.

**Additional file 5: Supplementary Table 3.** Results of univariate analysis of periodontitis in men. 

## Data Availability

The datasets generated and analyzed during the current study are available in the NHANES repository: NHANES—About the National Health and Nutrition Examination Survey (cdc.gov).
