# Supplementary material for: Association between serum uric acid, hyperuricemia and periodontitis: a cross-sectional study using NHANES data
Source: BMC Oral Health. 2023 Aug 30;23:610. doi: 10.1186/s12903-023-03320-4 (PMC10466695; doi:10.1186/s12903-023-03320-4)
Supplement: Supplementary file 2 — Additional file 2: Supplementary Figure 2. Subgroup analyses of SUA and periodontitis in the male population. Except for the stratification component itself, each stratification factor was adjusted for all other variables (age, race/ethnicity, education, marital status, income-poverty ratio (PIR), alcohol status, smoking status, dietary fiber, total fat, body mass index (BMI), gout, congestive heart failure, coronary heart disease, angina, stroke, weak/failing kidneys, diabetes, hypertension and dental visit). [file 12903_2023_3320_MOESM2_ESM.docx]

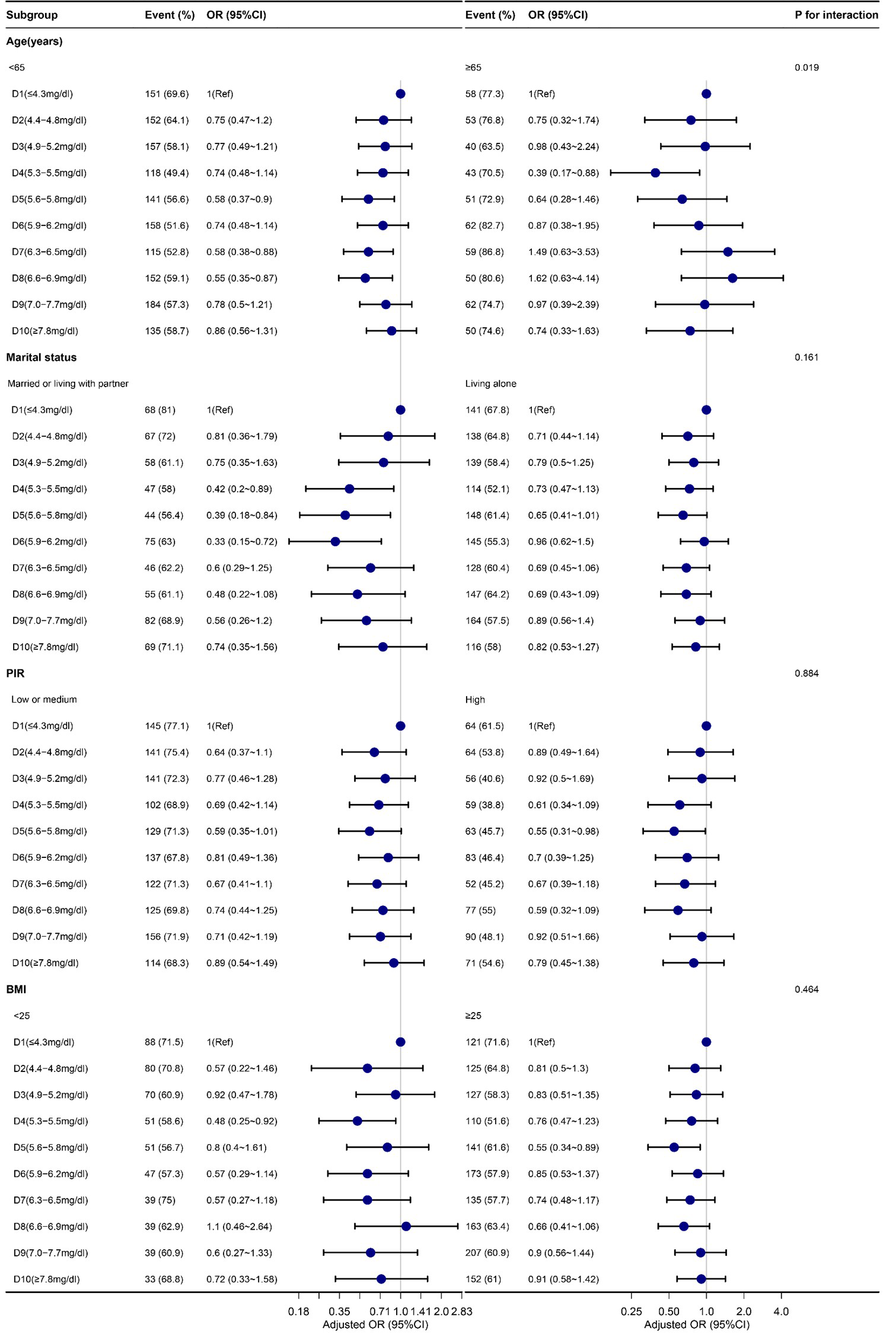


**Supplementary Figure 2** Subgroup analyses of SUA and periodontitis in the male population. Except for the stratification component itself, each stratification factor was adjusted for all other variables (age, race/ethnicity, education, marital status, income-poverty ratio (PIR), alcohol status, smoking status, dietary fiber, total fat, body mass index (BMI), gout, congestive heart failure, coronary heart disease, angina, stroke, weak/failing kidneys, diabetes, hypertension and dental visit).
