# Supplementary material for: Association between serum uric acid, hyperuricemia and periodontitis: a cross-sectional study using NHANES data
Source: BMC Oral Health. 2023 Aug 30;23:610. doi: 10.1186/s12903-023-03320-4 (PMC10466695; doi:10.1186/s12903-023-03320-4)
Supplement: Supplementary file 3 — Additional file 3: Supplementary Table 1. Baseline characteristics of the study population by sex. [file 12903_2023_3320_MOESM3_ESM.docx]

**Supplementary Table 1** Baseline characteristics of the study population by sex.

| **Variables** | **Total**  **(n = 6606)** | **Female**  **(n = 3369)** | **Male**  **(n = 3237)** | ***p*-value** |
| --- | --- | --- | --- | --- |
| Periodontitis, n (%) | 3484 (52.7) | 1493 (44.3) | 1991 (61.5) | <0.001^**^ |
| SUA (mg/dl) | 5.4 ± 1.4 | 4.9 ± 1.3 | 6.0 ± 1.3 | < 0.001^**^ |
| Hyperuricemia, n (%) | 1174 (17.8) | 539 (16) | 635 (19.6) | < 0.001^**^ |
| Age(years) | 51.9 ± 14.2 | 51.8 ± 14.1 | 52.0 ± 14.4 | 0.578 |
| Race/ethnicity, n (%) |  |  |  | 0.516 |
| Mexican American | 816 (12.4) | 402 (11.9) | 414 (12.8) |  |
| Other Hispanic | 630 (9.5) | 343 (10.2) | 287 (8.9) |  |
| Non-Hispanic White | 2711 (41.0) | 1376 (40.8) | 1335 (41.2) |  |
| Non-Hispanic Black | 1421 (21.5) | 724 (21.5) | 697 (21.5) |  |
| Non-Hispanic Asian | 858 (13.0) | 440 (13.1) | 418 (12.9) |  |
| Other Race | 170 (2.6) | 84 (2.5) | 86 (2.7) |  |
| Education, n (%) |  |  |  | < 0.001^**^ |
| <High school | 1372 (20.8) | 653 (19.4) | 719 (22.2) |  |
| High school | 1418 (21.5) | 690 (20.5) | 728 (22.5) |  |
| >High school | 3816 (57.8) | 2026 (60.1) | 1790 (55.3) |  |
| Marital status, n (%) |  |  |  | < 0.001^**^ |
| Married or living with partner | 2317 (35.1) | 1387 (41.2) | 930 (28.7) |  |
| Living alone | 4289 (64.9) | 1982 (58.8) | 2307 (71.3) |  |
| PIR, n (%) | 2.7 ± 1.7 | 2.6 ± 1.7 | 2.7 ± 1.7 | < 0.001^**^ |
| Alcohol status, n (%) | 4779 (72.3) | 2049 (60.8) | 2730 (84.3) | < 0.001^**^ |
| Smoking status, n (%) | 2855 (43.2) | 1143 (33.9) | 1712 (52.9) | < 0.001^**^ |
| Dietary fiber (gm) | 15.6 (10.2, 23.1) | 14.2 (9.4, 20.6) | 17.4 (11.3, 25.5) | < 0.001^**^ |
| Total fat (gm) | 72.2 (48.3, 102.5) | 64.1 (43.4, 89.8) | 82.2 (55.5, 114.5) | < 0.001^**^ |
| BMI (kg/m^2^) | 29.2 ± 6.8 | 29.8 ± 7.6 | 28.7 ± 5.7 | < 0.001^**^ |
| Gout, n (%) | 267 (4.0) | 75 (2.2) | 192 (5.9) | < 0.001^**^ |
| Congestive heart failure, n (%) | 164 (2.5) | 86 (2.6) | 78 (2.4) | 0.709 |
| Coronary heart disease, n (%) | 196 (3.0) | 65 (1.9) | 131 (4) | < 0.001^**^ |
| Angina, n (%) | 138 (2.1) | 62 (1.8) | 76 (2.3) | 0.149 |
| Stroke, n (%) | 184 (2.8) | 98 (2.9) | 86 (2.7) | 0.534 |
| [Weak/failing kidneys](https://wwwn.cdc.gov/Nchs/Nhanes/2011-2012/KIQ_U_G.htm#KIQ022), n (%) | 213 (3.2) | 109 (3.2) | 104 (3.2) | 0.959 |
| Diabetes, n (%) | 849 (12.9) | 413 (12.3) | 436 (13.5) | 0.142 |
| Hypertension, n (%) | 2526 (38.2) | 1300 (38.6) | 1226 (37.9) | 0.551 |
| Dental visit, n (%) | 3912 (59.2) | 2100 (62.3) | 1812 (56) | < 0.001^**^ |

Abbreviation: SUA, serum uric acid; PIR, income-poverty ratio; BMI, body mass index.

**p* < 0.05; ***p* < 0.01.
