# Supplementary material for: Association between serum uric acid, hyperuricemia and periodontitis: a cross-sectional study using NHANES data
Source: BMC Oral Health. 2023 Aug 30;23:610. doi: 10.1186/s12903-023-03320-4 (PMC10466695; doi:10.1186/s12903-023-03320-4)
Supplement: Supplementary file 4 — Additional file 4: Supplementary Table 2. Results of univariate analysis of periodontitis in women. [file 12903_2023_3320_MOESM4_ESM.docx]

**Supplementary Table 2** Results of univariate analysis of periodontitis in women.

| **Variables** | **OR (95 %CI)** | ***p*-value** | **Variables** | **OR (95 %CI)** | ***p*-value** |  |
| --- | --- | --- | --- | --- | --- | --- |
| SUA (mg/dl) | 1.14 (1.08~1.21) | <0.001^**^ | 1-3 | 0.76 (0.63~0.91) | 0.003^**^ |  |
| Hyperuricemia (yes vs. no) | 1.45 (1.21~1.75) | <0.001^**^ | >3 | 0.32 (0.27~0.39) | <0.001^**^ |  |
| Age (years) | 1.04 (1.03~1.04) | <0.001^**^ | Alcohol status (yes vs. no) | 0.63 (0.55~0.73) | <0.001^**^ |  |
| Race/ethnicity |  |  | Smoke status (yes vs. no) | 1.46 (1.26~1.68) | <0.001^**^ |  |
| Mexican American | 1 (reference) |  | Dietary fiber (gm) | 0.99 (0.98~0.99) | <0.001^**^ |  |
| Other Hispanic | 0.67 (0.5~0.9) | 0.007^**^ | Total fat (gm) | 1 (0.99~1) | <0.001^**^ |  |
| Non-Hispanic White | 0.48 (0.38~0.6) | <0.001^**^ | BMI |  |  |  |
| Non-Hispanic Black | 0.96 (0.75~1.23) | 0.741 | <25 | 1 (reference) |  |  |
| Non-Hispanic Asian | 0.69 (0.53~0.91) | 0.007^**^ | 25–30 | 1.25 (1.05~1.5) | 0.014^*^ |  |
| Other Race/ethnicity | 0.51 (0.32~0.83) | 0.007^**^ | >30 | 1.51 (1.28~1.78) | <0.001^**^ |  |
| Education |  |  | Gout (yes vs. no) | 2.27 (1.41~3.66) | 0.001^**^ |  |
| <High school | 1 (reference) |  | Congestive heart failure (yes vs. no) | 2.82 (1.78~4.47) | <0.001^**^ |  |
| High school | 0.73 (0.59~0.91) | 0.005^**^ | Coronary heart disease (yes vs. no) | 2.68 (1.59~4.53) | <0.001^**^ |  |
| >High school | 0.35 (0.29~0.42) | <0.001^**^ | Angina (yes vs. no) | 2.32 (1.37~3.92) | 0.002^**^ |  |
| Marital status |  |  | Stroke (yes vs. no) | 1.86 (1.23~2.79) | 0.003^**^ |  |
| Married or living with partner | 1 (reference) |  | [Weak/failing kidneys](https://wwwn.cdc.gov/Nchs/Nhanes/2011-2012/KIQ_U_G.htm#KIQ022)(yes vs. no) | 2.42 (1.62~3.6) | <0.001^**^ |  |
| Living alone | 0.59 (0.51~0.67) | <0.001^**^ | Diabetes (yes vs. no) | 2.19 (1.77~2.7) | <0.001^**^ |  |
| PIR |  |  | Hypertension (yes vs. no) | 1.77 (1.53~2.03) | <0.001^**^ |  |
| ≤1 | 1 (reference) |  | Dental visits (yes vs. no) | 0.48 (0.41~0.55) | <0.001^**^ |  |

Abbreviation: SUA, serum uric acid; PIR, income-poverty ratio; BMI, body mass index.

**p* < 0.05; ***p* < 0.01.
