# Supplementary material for: Association between serum uric acid, hyperuricemia and periodontitis: a cross-sectional study using NHANES data
Source: BMC Oral Health. 2023 Aug 30;23:610. doi: 10.1186/s12903-023-03320-4 (PMC10466695; doi:10.1186/s12903-023-03320-4)
Supplement: Supplementary file 5 — Additional file 5: Supplementary Table 3. Results of univariate analysis of periodontitis in men. [file 12903_2023_3320_MOESM5_ESM.docx]

**Supplementary Table 3** Results of univariate analysis of periodontitis in men.

| **Variables** | **OR (95 %CI)** | ***p*-value** | **Variables** | **OR (95 %CI)** | ***p*-value** |  |
| --- | --- | --- | --- | --- | --- | --- |
| SUA (mg/dl) | 0.95 (0.9~1) | 0.051 | 1-3 | 0.66 (0.52~0.82) | <0.001^**^ |  |
| Hyperuricemia (yes vs. no) | 0.97 (0.81~1.16) | 0.745 | >3 | 0.28 (0.22~0.35) | <0.001^**^ |  |
| Age (years) | 1.04 (1.03~1.04) | <0.001^**^ | Alcohol status (yes vs. no) | 1.05 (0.86~1.27) | 0.63 |  |
| Race/ethnicity |  |  | Smoke status (yes vs. no) | 2.12 (1.83~2.44) | <0.001^**^ |  |
| Mexican American | 1 (reference) |  | Dietary fiber (gm) | 0.99 (0.99~1) | 0.01^*^ |  |
| Other Hispanic | 0.75 (0.54~1.04) | 0.089 | Total fat (gm) | 1 (1~1) | <0.001^**^ |  |
| Non-Hispanic White | 0.39 (0.3~0.49) | <0.001^**^ | BMI |  |  |  |
| Non-Hispanic Black | 1.01 (0.76~1.32) | 0.963 | <25 | 1 (reference) |  |  |
| Non-Hispanic Asian | 0.53 (0.4~0.71) | <0.001^**^ | 25–30 | 0.85 (0.71~1.01) | 0.068 |  |
| Other Race/ethnicity | 0.51 (0.31~0.82) | 0.006^**^ | >30 | 0.87 (0.72~1.04) | 0.128 |  |
| Education |  |  | Gout (yes vs. no) | 1.24 (0.91~1.68) | 0.174 |  |
| <High school | 1 (reference) |  | Congestive heart failure (yes vs. no) | 2.28 (1.33~3.93) | 0.003^**^ |  |
| High school | 0.65 (0.51~0.83) | 0.001^**^ | Coronary heart disease (yes vs. no) | 1.39 (0.96~2.03) | 0.085 |  |
| >High school | 0.25 (0.21~0.31) | <0.001^**^ | Angina (yes vs. no) | 1.37 (0.84~2.23) | 0.212 |  |
| Marital status |  |  | Stroke (yes vs. no) | 1.97 (1.2~3.24) | 0.008^**^ |  |
| Married or living with partner | 1 (reference) |  | [Weak/failing kidneys](https://wwwn.cdc.gov/Nchs/Nhanes/2011-2012/KIQ_U_G.htm#KIQ022)(yes vs. no) | 1.64 (1.06~2.54) | 0.025^*^ |  |
| Living alone | 0.78 (0.66~0.91) | 0.002^**^ | Diabetes (yes vs. no) | 2.17 (1.72~2.73) | <0.001^**^ |  |
| PIR |  |  | Hypertension (yes vs. no) | 1.52 (1.31~1.76) | <0.001^**^ |  |
| ≤1 | 1 (reference) |  | Dental visits (yes vs. no) | 0.43 (0.37~0.5) | <0.001^**^ |  |

Abbreviation: SUA, serum uric acid; PIR, income-poverty ratio; BMI, body mass index.

**p* < 0.05; ***p* < 0.01.
